# A Qualitative Study on Medication Taking Behaviour Among People With Diabetes in Australia

**DOI:** 10.3389/fphar.2021.693748

**Published:** 2021-09-20

**Authors:** Akram Ahmad, Muhammad Umair Khan, Parisa Aslani

**Affiliations:** The University of Sydney School of Pharmacy, Faculty of Medicine and Health, Sydney, NSW, Australia

**Keywords:** medication taking behaviour, medication adherence, type 2 diabetes, indian migrants, factors, three phases of adherence

## Abstract

**Background:** Australia has a high proportion of migrants with an increasing migration rate from India. Type II diabetes is a long-term condition common amongst the Indian population.

**Aims:** To investigate patients’ medication-taking behaviour and factors that influence adherence at the three phases of adherence.

**Methods:** Semi-structured interviews were conducted with a convenience sample of 23 Indian migrants living in Sydney. All interviews were audio-recorded, transcribed verbatim and thematically analysed.

**Results: *1) Initiation:*** The majority of participants were initially prescribed oral antidiabetic medicine and only two were started on insulin. Most started taking their medicine immediately while some delayed initiating therapy due to fear of side-effects. ***2) Implementation*:** Most participants reported taking their medicine as prescribed. However, some reported forgetting their medicine especially when they were in a hurry for work or were out for social events. ***3) Discontinuation:*** A few participants discontinued taking their medicine. Those who discontinued did so to try Ayurvedic medicine. Their trial continued for a few weeks to a few years. Those who did not receive expected results from the Ayurvedic medicine restarted their prescribed conventional medicine.

**Conclusion:** A range of medication-taking behaviours were observed, ranging from delays in initiation to long-term discontinuation, and swapping of prescribed medicine with Ayurvedic medicine. This study highlights the need for tailored interventions, including education, that focus on factors that impact medication adherence from initiation to discontinuation of therapy.

## Introduction

Diabetes mellitus (DM) is a set of metabolic disorders characterised by hyperglycaemia, caused by a lack of insulin release as well as its activity (Diabetes Australia, 2018). There are several types of diabetes, with the two most prevalent types being type 1 (T1D) and type 2 diabetes (T2D) (Diabetes Australia, 2018). T1D (also known as insulin dependent or juvenile diabetes) is characterised by lack of insulin production by the pancreas and accounts for 5–10% of total cases (American Diabetes Association, 2014; Diabetes Australia, 2018; IDF Diabetes Atlas, 2019). T2D (also called non-insulin-dependent diabetes) occurs due to inadequacy of the body to effectively utilize insulin (IDF Diabetes Atlas, 2019). T2D accounts for the majority of cases (85–90%) around the globe (Diabetes Australia, 2018). As the research presented in this article explores understanding of T2D management in Indian migrants in Australia, only issues related to T2D have been presented.

Diabetes is a long-term, progressive medical condition that requires continuous monitoring and management (Low et al., 2016). The main goals of diabetes management are to reduce symptoms, prevent associated complications and improve quality of life (Low et al., 2016). Patients can achieve these goals by adhering to their medicine (Krapek et al., 2004), lifestyle modifications such as a healthy diet, and regular exercise to maintain optimal blood sugar levels and body weight (Chang et al., 2007; Naja et al., 2014). However, poor medication adherence in T2D is well documented to be very common, and the clinical burden of non-adherence to antidiabetic medication is associated with inadequate glycemic control; increased morbidity and mortality; and increased costs of outpatient care, emergency room visits, hospitalization, and managing complications of diabetes (Polonsky and Henry, 2016). The incidence of anti-diabetic medication adherence has been reported to range between 38 and 93% (Polonsky and Henry, 2016). For example, in the United Arab Emirates, Ethiopia, Uganda, Switzerland, Botswana, and India medication adherence has been reported as 84% (Arifulla et al., 2014), 85.1% (Abebaw et al., 2016), 83.3% (Bagonza et al., 2015), 40% (Huber and Reich, 2016), 52% (Rwegerera et al., 2018) and 82.4% (Basu et al., 2018), respectively. Dhippayom et al. (2015) conducted a study among Australian patients with type 2 diabetes and found that adherence to anti-diabetic medicines was suboptimal at 64.6% (Dhippayom and Krass, 2015).

In Australia, T2D is a leading cause of morbidity and mortality and approximately 1.2 million people have been diagnosed with T2D (Diabetes in Australia, 2020). According to the Australian Bureau of Statistics as of June 2019, approximately 2.6% of the total Australian population are migrants from India (Australian Bureau of Statistics, 2020). There is a very high prevalence of diabetes among Indian-born migrants (14.8%) compared with the Australian-born population (7.1%) (Centre for Epidemiology and Research, 2006), with a higher rate of hospitalisation due to diabetes and its complications (New South Wales Health, 2008). Indian migrants may face unique healthcare challenges. Maintaining normal blood sugar levels may not be easy among Indian migrants because of several other factors affecting their health. For example, an unhealthy diet (Wells et al., 2016), poor physical activity (Venkatesan et al., 2018), poor medication adherence (Venkatesan et al., 2018), poor awareness about the health system (Straiton and Myhre, 2017), high costs of treatment (Grattan Institute, 2021), stress related to migration (Chiarenza et al., 2019), finding a job, and other family related issues (Worthington and Gogne, 2011). Furthermore, their cultural and religious beliefs may also affect their medication-taking behaviour (Worthington and Gogne, 2011).

Vrijens et al. (2012), categorised adherence into three phases, initiation, implementation, and discontinuation. “*Initiation*” was defined as “*when a patient takes the first dose of prescribed medication*”. Initiation is followed by “*implementation*”, and this relates to “*the extent to which a patient’s actual dosing corresponds to the prescribed dosing regimen*”. “*Discontinuation*” is “*when the next dose to be taken is omitted and no more doses are taken thereafter*” (Vrijens et al., 2012). Understanding each phase is important in identifying and exploring effective ways of improving adherence.

While previous research indicates that many contributing factors have yet to be identified (Krass et al., 2015), this could be due to factors affecting different populations and factors influencing the three stages of adherence. The majority of studies in the literature either have not disclosed the phase of adherence studied (Hugtenburg et al., 2013; Sapkota et al., 2017) or did not investigate adherence at a specific phase. Moreover, the studies mentioned above which have reported adherence rates, have not necessarily reported rates according to the three phases of adherence.

While adherence to anti-diabetic medicines in the Australian community was found to be suboptimal (Dhippayom and Krass, 2015), there are limited studies on adherence and medication-taking behaviour among the migrant population in Australia. To date, no study has attempted to understand anti-diabetic medication-taking behaviour among Indian migrants with diabetes in Australia. Therefore, this in-depth qualitative study aimed to investigate patients’ medication-taking behaviour and factors that influence adherence, particularly at its three phases.

## Methods

### Qualitative Research

Qualitative studies provide a rich exploration of individuals’ experiences and perspectives (Holloway, 1997). Qualitative methodology is valuable in developing a deeper understanding of the behaviour of patients taking anti-diabetic medicines and the factors that influence their medication taking, as quantifying the findings does not fully capture individuals’ experiences. This study was part of a larger research project that investigated the medication taking behaviour of Indian migrants with type 2 diabetes living in Sydney, Australia. This paper only reports on the findings of the questions about participants’ medication-taking behaviour and factors that influence adherence.

### Research Questionnaire

The semi-structured interview questionnaire addressed the research aim of investigating Indian migrants’ (with type 2 diabetes) understanding of the Australian healthcare system, their medication-taking behaviour, and disease management. The questions were developed based on previous research (Ching et al., 2013; Ahmad et al., 2015; Namageyo-Funa et al., 2015; Zainudin et al., 2017; Sapkota et al., 2018; Garad and Waycott, 2015). In-depth, face-to-face interviews were conducted with eligible participants at public places in Sydney, Australia. The semi-structured questionnaire was tested for its content and face validity with four participants, consisting of two qualitative methods researchers acting as pseudopatients, one member of the public (Indian migrant) and one Indian migrant with type 2 diabetes. Only minor wording changes were made before the actual interviews were conducted. At the beginning of the interview, the participants were provided with the Participant Information Statement to ensure that they had had the opportunity to read the information and ask questions, in addition to when they received the PIS at recruitment. Participants were also asked to complete a brief questionnaire on demographic details, and the Summary of Diabetes Self-Care Activities (SDSCA) (for adherence data).

### Ethical Approval and Participant Recruitment

This study was approved by the Human Research Ethics Committee (2018/415) of The University of Sydney. Participants were recruited within Greater Sydney and its surrounding suburbs, using three approaches. Firstly, advertising was conducted *via* Facebook, Gumtree, and Indian news media e.g., Indian Link. Secondly, Indian organisations and associations were approached to post the study flyer on their social media channels and distribute among their members. Thirdly, passive snowballing was used *via* the participants. Interested participants contacted the researcher directly. The researcher then provided further information about the study (a copy of the Participant Information Statement (PIS) which included detailed information about the study, and other issues such as anonymity, confidentiality and voluntary nature of participation in the study), and if the person enquiring was interested in participating, assessed them for eligibility before discussing an interview time and location. All participants were provided with study information in an unbiased manner so that no coercion was perceived by the potential participants.

Participants were included in the study if they were:

• 18 years or above
• diagnosed with type 2 diabetes and using at least one anti-diabetic medicine
• Indian born migrant living in Sydney as an Australian citizen or as a permanent resident
• not dependent on others to administer their medicine
• fluent in English or Hindi.

Written consent was obtained from each participant before the interview was started. The interviews lasted for about 40–45 min. The interviews were audio-recorded. All interviews, except one (conducted in Hindi), were conducted in English directed by the PhD student (AA) who is a registered pharmacist in India and trained in qualitative research techniques. The data saturation point was defined as the point after which no new data was acquired. Saturation was accomplished by the 18th participant and a further five interviews were conducted to ensure confirmation of identified themes. Each participant was reimbursed AUD 30 for their time and travel expense.

### Data Analysis

The interview recordings were transcribed verbatim and thematically analysed by AA using a framework for thematic analysis (Spencer et al., 2003). The first four interview recordings were transcribed and analysed by AA and reviewed by PA. The remainder of the interview recordings were transcribed through a NAATI (National Accreditation Authority for Translators and Interpreters) certified transcription company. Each transcript was rechecked by AA and inconsistencies were identified and corrected. All transcripts were de-identified, and the process was completed by removing all personal information from the participants’ transcripts. Data analysis was performed manually and recorded manually using Microsoft Word. Initially, the two researchers (AA and PA) independently went through some of the transcripts to gain meaning from the discussions, generated codes and derived themes. The process began as AA read the transcripts line by line and listened to the audio recordings multiple times to become familiar with the data. The initial codes were generated by AA through inductive coding. For transcript coding, a latent technique was used to capture the logical and underlying meaning of the findings. Then, important statements were identified related to the beliefs, decision-making process and experiences of the participants. After that, meanings were formulated from selected statements. Every meaning was coded and the process of combining the codes into a concept resulted in a number of themes. Finally, we combined themes to reflect the study objectives. The research team met regularly to discuss the nodes, codes, and themes generated from the transcripts.

The consolidated criteria for reporting qualitative research (COREQ) was used to provide transparency in data reporting to improve rigour, comprehensiveness and trustworthiness of the study (Tong et al., 2007).

Adherence was assessed using the Summary of Diabetes Self-Care Activities (SDSCA) was calculated based on the following formula (Toobert et al., 2000). “% Adherence Score = Sum of adherence score for each anti-diabetic medication/(7*number of medications)*100%. Adherence score for each anti-diabetic medication = (7 − number of days medications were missed).”

## Results

### Demographics

Twenty-three participants were interviewed. The majority of participants were male (*n* = 18). Eight participants were diagnosed within the last 5 years. Sixteen participants were taking at least one oral anti-diabetic medicine and two participants were on insulin (Table 1).

**TABLE 1:** Demographic information of participants.

Characteristics	Variables	Data
Age (years)	Median	39
	Range	33–72
Gender	Male	*n* = 18
	Female	*n* = 5
Duration of living in Australia	<5 years	*n* = 8
	≥5–10 years	*n* = 7
	>10 years	*n* = 8
Duration since diagnosis	Median	5 years
	Range	4 months—40 years
Types of antidiabetic medicines	Oral	*n* = 16
	Oral + insulin	*n* = 0
	Insulin	*n* = 2
	Oral + Ayurvedic medicine	*n* = 3
	Ayurvedic medicine	*n* = 2

### Assessment of Medication Adherence Using Summary of Diabetes Self-Care Activities

Five of the 23 participants reported having missed at least one dose of their anti-diabetic medicine in the past 7 days. The average adherence score (%) calculated using the SDSCA questionnaire was 97.2% (Table 2).

**TABLE 2:** Assessment of medication adherence using SDSCA (*n* = 23).

Data on anti-diabetic medicines	Data
Minimum number of medicines	1
Maximum number of medicines	4
Median	2
Average	1.60
Adherence assessment SDSCA	—
Minimum score (%)	71.4
Maximum score (%)	97.2
Average score (%)	92.9

### Qualitative Findings

The interview findings were categorised into three broad themes, focusing on medication-taking behaviour and factors that influence adherence at the three phases of adherence: initiation, implementation, and discontinuation. The factors influencing adherence were separated for each phase and grouped into facilitators and barriers.

Participants reported that they were diagnosed with diabetes either as a result of a routine health check-up (*n* = 6); when they suspected to have a diabetes-related symptom(s) and proceeded to self-test in India (*n* = 8); or when they went to see their doctor for non-diabetes-related complaints/symptoms (*n* = 12), or a combination of the above (*n* = 3). They initiated prescribed medicine alongside self-medication such as with Ayurvedic medicine (AM) and/or supported by religion and spirituality. The findings on AM and the impact of religion and spirituality have been presented elsewhere (Ahmad, 2021).

### Medication Taking Behaviour and Practices

The majority of participants initiated their therapy soon after their diagnosis while some participants tried Ayurvedic medicines that were brought from India, initially with or without conventional medicine. If no benefits were seen in their blood sugar levels after using the AM, participants went back to the prescribed regimen. Once patients made their decision to adhere to the prescribed medicines, they felt and reported that there was no difficulty in adhering to the medicine.

#### Initiation

The majority of participants were initially prescribed an oral antidiabetic medicine and only two were started on insulin. Five participants were first advised of using non-pharmacological approaches such as physical exercise and a restricted diet before they were prescribed an oral antidiabetic medicine. The majority of the participants started taking their prescribed medicine, while some were in a dilemma whether to start because of various concerns such as long-term use and side-effects.

##### Facilitators for Initiating Prescribed Medicines

###### Desire to Improve Diabetes Outcome

Most of the participants reported that the key facilitator for initiating medicines was that they understood the importance of medicines in improving diabetes outcomes. They wanted to control their blood sugar level and knew that if they did not start, their blood sugar level could increase further and create problems. Importantly, they reported that starting their medicine reduced the chances of future diabetes-related complications.

“I know very well about diabetes, it’s a very dangerous disease, it will affect my organs like my kidney, eye etc, so you need to control it and it can only be managed with medications” [PD 9]

##### GPs Recommendations and Being Informed About Medicines

Most of the participants reported that GP consultations and their advice was an important factor in motivating them to initiate medicines as soon as they were diagnosed with diabetes. There was a clear disparity in participants’ awareness of diabetes and its pharmacological and non-pharmacological treatment options, as well as the information they had received from their GPs. Those diagnosed in Australia tended to receive information from their GPs to increase their knowledge about diabetes and its treatments, and to reduce their fear and misconceptions about the medicine. This supported the participants to initiate medication taking. Additionally, these participants reported that they received very good diabetes-related services in Australia, which they reported were as a result of their visit to the GP. These services included a health plan, dietician and eye testing, which they felt better supported their diabetes treatment and care. In contrast, the experience of those who were diagnosed in India was regarded as far from optimum. Participants reported that the doctors only prescribed the medicine, and they had to pay for all other services themselves or through private insurance. They felt that they were not educated about their condition or medicine. Participants reported that they at first did not agree to use the medicine as a result of lack of understanding, but that they later decided to initiate the prescribed medicine after they had been informed either by receiving information or having searched for information themselves.

*“In my opinion, you need to trust your doctor and follow their advice, that’s what I’ve learned from my experience.”* [PD 3]

*“I’m lucky to [be] diagnosed in Australia, when I got diagnosed with diabetes, I got a healthcare plan … I got four sessions with a dietitian and eye check up to plan out my wellbeing, that kind of thing, so yeah it’s good...but in case of India, its totally opposite, I have to pay for everything and public healthcare system totally bad over there, I never use it, I always go to my private doctor”* [PD 7]

*“I was diagnosed with blood donation camp in India, but my sugar was high later on when I was prescribed [Brand X] [metformin], no other information provided... I wasn’t ready to start medication, I wasn’t comfortable with a lack of understanding about diabetes and medications, but I started looking for diabetes, videos on Youtube, I did my own research, and my knowledge grew and was more comfortable.”* [PD 17]

##### Negative Beliefs About Medicines

Negative beliefs about medications was an important barrier to initiating therapy. The fear of side effects and medication dependence were the main factors mentioned by the participants.

However a few, despite the prescription issued by their GP, had delayed their initiation. The period of the delay ranged from a few months to a few years. They opted to start with some alternative therapies, such as home remedies, ayurvedic medicine or herbs.

*“My colleague in Australia said allopathic medications [conventional medicines] have lots of side effects, you need to use it [for a] lifetime, she insisted to take ayurvedic medication because her mother using it and benefitted in India, … , so I decide to initiate ayurvedic medication instead of metformin as prescribed by my GP, I give try to use it, and found it effective with no side effects … I check the glucose level time to time … the name Sakkarai Kolli.”* [PD 22]

##### Fear of Injection, Discomfort and Pain

Three patients had used insulin in the past and two were currently using insulin. One patient was initiated with insulin due to high blood sugar levels. Another participant started using oral medicine and then moved to insulin, but the patient initially did not want to use insulin because of the need to inject regularly, the fear of pain, and injection scars. This resulted in a delayed use of insulin but the patient eventually started using insulin when their diabetes was not controlled with oral medication alone.

*“Initially my doctor prescribe metformin but later consultant want to change it to start insulin due to my sugar level high but I do not want to use Insulin because it is injection, right, I need to use it daily, it gives ‘dard’ [pain] and if use injection daily scars will appear … I’m afraid of injection from my childhood”* [PD 23]

#### Implementation

The majority of participants reported that they adhered to their prescribed medicines, however, they were unintentionally non-adherent through forgetting to take their medicine, especially when they were in a hurry to get to the office or when they were out at family dinners or parties.

##### Benefits of Antidiabetic Medication

Most reported having experienced the benefits of taking antidiabetic medicine which strengthened their confidence in the importance and need to take the medicine. Participants stated that they were more likely to continue using medicines that they have benefited from. However, what they considered as advantages of the medicine differed between the participants. For example, improved blood glucose levels and health fitness, were two positive outcomes reported by the participants.

*“My GP prescribed metformin initially and [I] start taking medication and it control my sugar….improves blood glucose level and also feel physically fit”* [PD 1]

##### Intentional and Non-intentional Missed Doses

The majority of participants said that medication-taking had become a habit for them and they did not tend to forget to take their medicine. There were some who mentioned that they sometimes forgot to take their medicines because medication-taking was not an established part of their daily routine as yet. They were sometimes reminded by their family members or they used their mobile phone alarm as a reminder. Some participants did take their medicines as soon as they were reminded, while others skipped the dose and decided to continue with the next dose. Some participants also reported that they did not completely adhere to the prescribed regimen as they may miss a dose or there may be delays in their meal times due to religious and cultural rituals, festivals, etc. Sometimes unplanned social gatherings also influenced the timing of their meals and affected the timing of medication use, and sometimes this resulted in a missed dose.

Participants’ behaviour indicated that adherence to prescribed medications was intentionally and non-intentionally non-adherent.

*“Initially I may have been forgotten, but now it’s in my everyday work routine. Usually 99% [of the time I] don’t forget to take medication, but sometimes forgot about it because of some weird scheduling, you have to do something else, or you’ve gone out or you’re out of town, xyz.”* [PD 9]

Some participants reported to have not adhered to their prescribed medicine because they did not want to take medicine or felt that they were careless about looking after their diabetes due to a lack of knowledge and motivation to adhere.

*“First few periods, I was very reluctant, forget. My problem was I was quite rebellious to take it in precise. Nothing’s going to happen to my body, I’m fit and fine, actually I was careless. I was not fully aware about diabetes and its future consequences. Over the period I read, study about diabetes and also my wife remind and motivate me, taking these medications.”* [PD 11]

Some participants said they used a pillbox (e.g.,Webster pack) to help them take their medicine on time. One of the participants said that she kept her pillbox next to the bottle of water as a reminder for her to take her medications whenever she went to drink water.

*“Nowadays I am using medication box, I keep for one week or one month some times, …. If you see the box is empty … So it’s a way to remind you.”* [PD 5]

Some participants shared their strategy of dealing with forgetfulness, especially if going out. To avoid forgetting to take their medicines, they ensured that they always carried their medicines with them, especially when going to social gatherings. One female participant noted that it was challenging to remember to take medicines when at social gatherings. She mentioned that she always carries her bag with necessary medicines, jelly beans and some fruits.

*“Whenever I got invited [to] any social or religious gatherings, first thing is I will ask, ‘What time you will give dinner?‘…they reply ‘We will give you food at 7.30’… I will have my medication planning, … I need to take my insulin, jellies and some fruits, so if needed or late so I can take accordingly … if they delay I will go straight to the kitchen and I will ask, “Who is responsible for this food.”* [PD 21]

##### Fear of Side Effects and Medication Dependency

Once they started taking medication, the key concerns were around side effects and addiction which negatively influenced medication use and adherence. The majority of participants held the belief that conventional medicines have side effects and once they started taking medicines they would not be able to stop, which led them to continue and remain dependent on their medicine. Participants’ reported that diabetes is a long-term condition and that patients have to rely on taking medicines for all of their lives.

*“Taking medication … want control my sugar as soon as possible, My plan is that I am going to be on this medication only for 1–2 years. Want to be away from all these strong drugs [because of side effects] and make addicted, do not want dependency on the medication, need to avoid as much as can … going to start natural method such as ayurvedic medication”* [PD 19]

##### Stigma Related to Diabetes

Many participants, however, delayed taking their medicines when they were out as they were not comfortable letting others know that they were diabetic. This feeling of discomfort also extended to their diet and showing anything different that would identify that they may be diabetic. For example, when taking tea or coffee, they did not inform people that they did not take sugar because of their diabetes. Participants mentioned that they made up for skipping a dose by increasing the next dose of their medicine.

*“A lot of Indian Diabetic patients and in social or religious getherings, nobody will open their mouth for example they need tea without sugar…..in Indian culture after food provide sweets. If someone offer tea, sweets, or anything no one refuse, even myself initially for few years, use the same and its put extra calorie in their diet … they always said “Oh! I will take one more tablet now I did not take and straight away say to them, I am diabetic”* [PD 21]

Participants raised concerns about the social stigma associated with diabetes, especially young patients with diabetes who did not disclose to others about their condition. They believed that the stigma negatively affected their dietary habits, scheduling appointments with a physician, and medication-taking behaviour (delayed or postponed medication use).

*“Initially I met my GP in Australia and [was] diagnosed with type 2 diabetes and started with metformin but I did not inform my wife, and even to my parents that I am diabetic, So I do not take medicine in front of her [wife]…I am young, just 36 year old…. later inform only wife and father … my mother doesn’t know, untill later and any of relatives or my friends, colleagues … wherever we go in social or religious gatherings, I used to take same food as no one knew that I am diabetic … the problem in Indian community they will show pity, “OMG you are ‘beemar’ [ill person]”..they will give advice take this and that to cure diabetes that’s why I do not want let others know I have diabetes. For my wife, initially, did not inform, because it would put fear in her…. I feel guilty of not seeing a regular GP and not taking medicine on time.”* [PD 7]

#### Discontinuation

This theme provides an overview of why participants discontinued their medicines.

##### Fear of Medication Side-effects

Fear of side effects was also identified as a common barrier to persistence with medication use, and therefore, a key facilitator of medication discontinuation. Only three participants had discontinued their conventional medicine because of the fear of experiencing side effects. They had commenced ayurvedic medicine instead as they believed it was made up of natural herbs, and hence, free of any side-effects.

It was noticed that participants discontinued and then restarted the medicine a few times in their medication-taking journey. For example, one participant discontinued his medicine a few times due to fear of side effects and started taking ayurvedic medicine. When he visited India (usually on vacation every year), he stopped his prescribed conventional medicines and sought some ayurvedic medicine as suggested by his brother/relatives as they believed that it would be effective to regulate his blood sugar levels and was free from side effects.

*“Initially insulin start (sugar was high) …. continued …. When it was normal after 4–5 months and few times went to India as it was suggested by my brother to take ayurvedic medication, he told that its really effective and no side effects.”* [PD 2]

Another participant reported he discontinued his prescribed medicine for a month to try ayurvedic medicine, but resumed taking the prescribed medicine when he did not experience any benefits with the ayurvedic medicine.

*“Immediately start metformin, later it was under control … my friend brought from India ayurvedic medication he was a diabetic and using it … said like its very effective, its made with all-natural ingredients, but no I didn’t see much benefit out of it, I used it for 1 month... my friend told me that if we used this ayurvedic medication on a regular basis, diabetes would be gone completely. My sugar level was still high so again back to my medication that was prescribed by my GP.”* [PD 14]

One other participant reported that he discontinued his medication for 1 year and managed his diabetes through his lifestyle changes such as restricted diet, exercise, and meditation.

*“I was diagnosed in 2002, since then using prescribed medications but later stop taking medication because I know predicted bad consequences in later life. I believe diabetes is a condition due to bad lifestyle and need to change it … no need to take medication … 1* *year no medication taking and manage well with his healthy diet, exercise and meditation with some herbs.”* [PD 4]

## Discussion

To the best of our knowledge, this is the first study conducted among Indian migrants with type 2 diabetes in Australia that explored their medication-taking behaviour and factors affecting their adherence to prescribed anti-diabetic medicine. The findings of this study provide insight into the participants’ medication-taking behaviour. The findings revealed that once a patient understood the need for a medicine to control their diabetes, they adhered better to their medicine; however, some discontinued and restarted their medicine due to fear of side-effects, medication dependency, and also to try ayurvedic medicine to manage their diabetes.

The qualitative nature of this study allowed for the exploration of participants’ medication-taking behaviour and how it changed from the initiation to discontinuation phase. Initiation of medicine occurred after the participants were diagnosed with diabetes and prescribed their medicine. At the initiation phase, most participants recognised that there were some health problems related to diabetes which needed to be resolved by medicines. Once they were diagnosed and prescribed a medicine by the physician, most participants initiated their therapy. Participants’ decision to initiate medicines was motivated by their belief in antidiabetic medicines and their desire to improve their health outcomes of diabetes.

An interesting finding of our study was the disparity reported by the participants who were diagnosed in India and Australia about awareness of diabetes and its management (pharmacological and non-pharmacological). Those diagnosed in Australia, showed a good understanding of diabetes and its medicines as they received information from their GPs when they were diagnosed to increase their knowledge about diabetes and its treatments, and reduce their fear and misconceptions about the medicines. These were the key facilitators for the initiation and continuity of anti-diabetic medicines. On the other hand, those diagnosed in India reported that they were only given a medicine by the doctor, and no other information was provided to them, and they thought that they had not been informed about their condition or medicine, which meant that their knowledge about diabetes and its therapy was sub-optimal in their opinions. Because of their poor understanding, they did not agree to initiate therapy and postponed medication taking for some time. Knowledge was therefore important in medication taking decision making. Similar findings have been reported in the literature, where those diagnosed with diabetes in India, have shown poor knowledge of diabetes, medicines and its prevention methods (Murugesan et al., 2007). Due to this lack of knowledge, they were reluctant to screen for further complications related to diabetes (Agarwal et al., 2005). Further, another study demonstrated poor awareness and hesitancy among newly diagnosed people with diabetes to screen for the retinopathy (11.6%) (Agarwal et al., 2005). These findings indicate a lack of adequate education regarding diabetes among the general public and also in diabetic patients especially those diagnosed in India. Therefore, serious efforts are required to educate patients about prevention and complications related to diabetes especially when they are first seen in an Australian GP clinic after arriving in Australia, or when first diagnosed in Australia.

The negative beliefs about medications (barriers) were reported by participants e.g., side effects, insulin use related discomfort, which delayed consultation with a doctor or delayed initiating prescribed conventional medicines regardless of their high blood sugar. During this period (when delaying or not taking their conventional medicine), participants tried some ayurvedic medicines and/or lifestyle modification and evaluated those strategies for any harmful or beneficial effect on their condition. Delay in seeking help from the healthcare providers or the initiation of their treatment was also reported by (Sapkota et al., 2017). Lack of knowledge about the actual side effects of diabetes medications may have contributed to the negative beliefs (Sapkota et al., 2017). Previous research has also identified that a lack of knowledge about diabetes medicines can lead to misconceptions about the medicines. Some participants who believed that the medication would harm them, delayed initiation of therapy. This finding was also reported by Polonsky and Henry, (2016). Some participants used insulin and initially did not want to initiate insulin use because they needed to inject regularly, and this caused anxiety and fear about the pain and discomfort caused by the needle. Studies have revealed that anxiety and fear of injection-associated pain have been reported to affect approximately 30–50% of patients initiating insulin (Kruger et al., 2015).

The physician-patient relationship was also identified as an important factor impacting medication adherence, especially in the transition from initiation to the implementation phase. In this study, those who were diagnosed in Australia showed a good physician-patient relationship and communication which led to a better understanding of diabetes and medications. Previous research indicates that a good relationship with a GP (physician) may have a positive effect on a person’s decision to initiate therapy (Chipidza et al., 2015). Where diet and exercise have not been effective in controlling a person’s blood glucose levels within the target range, early initiation of antidiabetic medicines is important for effective diabetes control (Diabetes Australia, 2018). Delaying initiation of therapy can affect the general control of blood sugar levels. GPs must identify those patients that may delay medication taking and help them make informed decisions about initiating medicines to improve their health outcomes.

### Implementation and Discontinuation Phase

In this study, once patients decided to initiate the medicine, they tended to continue with their medication, and continuation appeared to be influenced by their experience with medication use and the benefits gained. The findings indicate that patients were actively weighing their benefits (positive experience) against their concerns (negative experiences) in deciding to continue with the medicine. Positive experiences were primarily affected by medication benefits such as improved blood sugar levels, and overall well-being, and the negative experience was attributed to fear of side effects, drug dependence and also stigma related to diabetes. Such results indicate that participants whose positive experience with medicines out-weighs the fear of possible negative experience, are more likely to continue and adhere to medicines during the implementation phase. At the implementation phase, further adherence to medications can be increased by reducing their diabetes-related stigma by physicians educating their patients on diabetes and medications. An example of the extent of stigma experienced was not letting anyone in the Indian community know about a person’s diabetes, which then led to non-adherence, in the form of skipping medications or delaying medication-taking when around other Indians in the community. This delay in medication-taking due to social community stigma appears to be an important factor among Indian migrants. They also did not seem to share information that they were diagnosed with diabetes even with their close relatives as they believed that people will pity them and start advising on controlling their diabetes. A review conducted on stigma related to diabetes in India showed that a person with diabetes is considered or branded as a sick person (Thanushiya et al., 2019). Social stigma towards people with diabetes affects several aspects of a person’s life and affects overall outcome (Capistrant et al., 2019). Therefore, there is a need to create awareness and educate people about diabetes and its medication, and related stigmas.

This study found that fear of side effects was the principal cause of medication discontinuation. The findings are similar to those reported by Thanushiya et al. (2019) in Sri Lanka which showed that 68% of patients were skipping their medicine due to fear of side effects (Gunathilake et al., 2017). Another study conducted among Indian and Pakistani migrants with diabetes in the United Kingdom reported poor adherence due to safety concerns (fear of side effects) (Lawton et al., 2005). Indians generally believe and/or think that conventional medications are required to be used for long-term which may be associated with side-effects. Once the blood sugar level is under control, it is common among patients to discontinue their prescribed medication and start self-medication with alternative treatment such as ayurvedic medicines. This behaviour may be due to a lack of understanding about diabetes and the importance of adhering to prescribed medication even when they feel better (Porqueddu, 2017).

Participants who decide to adhere to medicines usually trade-off between benefits and adverse effects (concerns) based on their belief and experience, demonstrating the supremacy of intentional non-adherence in the implementation to discontinuation phase (Horne et al., 2013). Addressing the perceptual barriers, such as effective management of side effects, can improve the overall experience of patients with their medication, which can help in preventing intentional non-adherence.

The present research supports previous results and incorporates other factors such as medication dependency, self-medication and available alternative medicines (such as ayurvedic medicine) as factors that affect medication cessation (discontinuation) (Sapkota et al., 2017). Participants reported that their concerns about medication dependency affected their decision to avoid taking medicines. There is little evidence on patients with diabetes and their beliefs about being dependent on their antidiabetic medicine. Participants became worried about their dependence that could lead to early medication discontinuation. Several approaches may be used for resolving patients’ fears about reliance on medicines and therefoe improving adherence, such as education about anti-diabetic medicines.

## Limitations

This study has some limitations such as a low number of women, and the fact that the findings of a qualitative study, by its very nature, cannot be generalised. Also, the study was in a population of Indian migrants in Australia, who were on at least one antidiabetic medication, and reported high adherence rates. The findings therefore may not be applicable to other Indian populations elsewhere.

## Conclusion

Medication-taking behaviour among Indian migrants changed, and was influenced by different factors, at the different phases of medication-taking. At the initiation phase, most started their conventional medicine as soon as prescribed by GPs, while some postponed treatment initiation. The decision to initiate and continue with medication taking was based on a balance between concerns and needs. The key motivation was the desire to improve diabetes outcome (control blood glucose level), and some participants were motivated by advice/recommendations from GPs and the information they received about the medicine to initiate treatment. Medication benefits continued to influence adherence during the implementation phase. Negative factors such as stigma and fear of side effects and drug dependence were barriers to adherence during the implementation phase. Potential side effects were a common barrier to medication at all three phases. A few participants discontinued taking conventional medicines once they started experiencing the benefits and moved to Ayurvedic medicines; however, they restarted conventional medicines if the desired results were not achieved with the Ayurvedic medicine. The existence of adherence phase-specific factors underlines the dynamic nature of medication adherence and indicates that adherence factors may change during the medication-taking journey. Therefore it is important to recognise phase-specific factors that can be the basis for effective and lasting interventions to enhance medication adherence.

## Data Availability

The raw data supporting the conclusion of this article will not be made available by the authors as the authors do not have approval from the Human Research Ethics Committee of The University of Sydney to release such data. Only de-identified group data is available.
