# Supplementary material for: A Qualitative Study on Medication Taking Behaviour Among People With Diabetes in Australia
Source: Front Pharmacol. 2021 Sep 20;12:693748. doi: 10.3389/fphar.2021.693748 (PMC8488297; doi:10.3389/fphar.2021.693748)
Supplement: Supplementary file 1 [file DataSheet2.docx]

**Appendix 2**: COREQ (Consolidated criteria for Reporting Qualitative research) Checklist

| **Item no.** | **Topic** | **Guide Questions/Description** |
| --- | --- | --- |
|  | **Domain 1: Research team and reflexivity** | |
|  | ***Personal characteristics*** | |
| 1 | Interviewer/ facilitator | Akram Ahmad |
| 2 | Credentials | BPharm, PharmD (Post Baccalaureate) |
| 3 | Occupation | PhD Candidate and Registered Pharmacist |
| 4 | Gender | Male |
| 5 | Experience and training | A registered pharmacist from India; with experience in teaching and research. Trained in qualitative techniques. |
|  | ***Relationship with participants*** | |
| 6 | Relationship established | Before each interview began, the researcher established relationship with the participant to the best of his ability. |
| 7 | Participant knowledge of the interviewer | Before starting an interview, each participant received a participant information statement (PIS) in their preferred language (English or Hindi) approved by the University of Sydney 's Human Research Ethics Committee, Australia.  The name and address of the researchers and their affiliation (Sydney University) was also included as part of the letter head appearing on the PIS document. Participants have knowledge about that. The researcher (name, affiliation, and role in the research) also introduced himself before each interview started. |
| 8 | Interviewer characteristics | PhD Candidate, academic researcher, and pharmacist. The researcher has remained to the best of our knowledge, Unbiased during the interviews. |
|  | **Domain 2: Study design** | |
|  | ***Theoretical framework*** | |
| 9 | Methodological orientation and Theory | No methodological orientation underpinned the study design. Thematic analysis was used to analyse the findings. |
|  | ***Participant selection*** | |
| 10 | Sampling | The convenience and snowball sampling used to locate the participants in Australia. The participants were recruited based on criteria set for the study for inclusion and exclusion. |
| 11 | Method of approach | Usage of different techniques to find the participants.  The methods have been described in qualitative methods section. |
| 12 | Sample size | 23 participants completed the study |
| 13 | Non-participation | 19 [initially 42 participants shows their interest to participate in the study, later some of them not available due to various reasons and later we reached saturation and no contact has been made further] |
|  | ***Setting*** | |
| 14 | Setting of data collection | Public venue suitable for the participant. |
| 15 | Presence of nonparticipants | In 18 interviews, only the participant and researcher were present. 2 participants were accompanied by spouse and 1 participant were accompanied her mother-in-law. |
| 16 | Description of sample | Indian born migrants living in Australia [ethnicity]. The detailed description given in qualitative methods. |
|  | ***Data Collection*** |  |
| 17 | Interview guide | The interview guide is shown in Appendix 1. |
| 18 | Repeat interviews | Repeated interviews were not performed and do not apply to this study. |
| 19 | Audio/visual recording | Audio recording was used to collect the data. |
| 20 | Field notes | Field notes were made right after each interview. |
| 21 | Duration | The mean average duration of the interviews was 42.45 minutes. |
| 22 | Data saturation | Data saturation was reached. |
| 23 | Transcripts returned | Transcripts have been transcribed verbatim and checked against audio to ensure accuracy prior to analysis. Transcripts were not returned for statement or correction to the participants. |
|  | **Domain 3: analysis and findings** | |
|  | ***Data analysis*** | |
| 24 | Number of data coders | AA and PA were analysed data using a framework for TA. The first four interview recordings were transcribed by AA and reviewed by PA. These four transcripts independently analysed by AA and PA; and the remaining analysed by (AA). The interviews recordings were fully transcribed and thematically analysed. |
| 25 | Description of the coding tree | The coding tree (diagram) is presented in qualitative method chapter (Figure 6.2) |
| 26 | Derivation of themes | Wide themes were used to build interview guide to answer research objectives. Specific themes developed from interviews, one of which appears here. |
| 27 | Software | Microsoft Word was used to manage the data. |
| 28 | Participant checking | Participants did not provide feedback on the findings. |
|  | ***Reporting*** | |
| 29 | Quotations presented | Representative quotations were presented while the findings were reported. |
| 30 | Data and findings consistent | There is consistency in the data and findings presented. |
| 31 | Clarity of major themes | Major themes are presented in Figure 6.2 (qualitative method) |
| 32 | Clarity of minor themes | Major themes are presented in Figure 6.2 (qualitative method) |
