# Supplementary material for: A Qualitative Study on Medication Taking Behaviour Among People With Diabetes in Australia
Source: Front Pharmacol. 2021 Sep 20;12:693748. doi: 10.3389/fphar.2021.693748 (PMC8488297; doi:10.3389/fphar.2021.693748)
Supplement: Supplementary file 2 [file DataSheet1.docx]

**Appendix 1: Qualitative study interview protocol**

**1. Interview protocol and data sheet- patient interviews**

*Patients will be informed verbally by the researcher about the study; will then be given the Participant Information Statement to read, and asked to read and sign the consent form, if agreeing to participate in the study. Participants will be encouraged to ask the interviewer if there is anything unclear, or if they need more information.*

*The process will then begin as follows:*

*Before we formally begin the interview, I would quickly like to ask you a few questions*:

**Participant’s Information and adherence assessment**

Patient no.: (*allocated by the interviewer for analysis purpose*): ……………

- Age…………….. Gender: …………; How long have you been in Australia?...
- Duration since diabetes diagnosis:……
- What other conditions do you have that you take or not take any medicines for?

……….

……….

……….

- Do you know the names of the medications that you are currently taking? (Y/ N)

………….

Can you tell me the name of your anti-diabetic medications?

1……………………………… ; 2. …………………………. ; 3……………………………….

4………………………………; 5. …………………………

Can you tell me the name of your other medications (if any) ? (Y/N) …………….

| 1…………………………… ; | 2. ………………..; | 3. .……………… |
| --- | --- | --- |
| 4. ……………………………; | 5. ………………..; | 6. ………………. |

Assessment of adherence Summary of diabetes self-care activity (SDSCA approach)^1,2^

On how many of the last 7 days did you take your recommended anti-diabetic pills? (*this will be asked for each anti- diabetic medication taken by the participant)*

Medicine 1: …… ; Medicine 2 : ….; Medicine 3: ……; Medicine 4:

Assessment of adherence (other medications- SDSCA approach)

On how many of the last 7 days did you take your other medications? ………

Do you find problem with any specific medication? Which one? ……………

**Interview questions:**

**Question 1:** What do you know about accessing the Australian healthcare system?

**Prompts;**

# *If you were sick or your family member, how would you go about getting help?*

# *From where can you get OTC medicines?*

# *If you had a minor illness, what would you do?*

# *If you had a more major issue, what would you do?*

# *If you have an emergency, where do you need to go?*

**Question 2:** When do you seek help for your diabetes illness?

**Prompts;**

- *Please share your experience about when you went to see your doctor?*
- *Regularly visit/irregular visit, why?*
- *When my diabetes uncontrolled?*

**Question 3:** How were you diagnosed with diabetes and how do you feel being diabetic?

**Prompts:**

- *Went to see doctor for other disease? Suspect symptoms of diabetes?*
- *How did you feel when the doctor first told you that you had diabetes?*
- *How has your feeling changed over time?*
- *How has diabetes and the medication(s) you take for diabetes affected your day to day living?*
- *How has diabetes and the medication(s) you take for diabetes affected your lifestyle in general?*

**Question 4:** Where and from whom, do you get information about your diabetes and medications you take for diabetes?

**Prompts;**

- *In your day to day life where you get information about diabetes and its medications? (healthcare professional in hospital, community pharmacy, internet, media or other)*
- *Do you think that information in Hindi is more useful to you compared to in English*

*Anything you would like to suggest to improve health information*

**Question 5:** What are some of your difficulties and facilitators (motivators) for you for seeking diabetic care in Australia?

**Prompts;**

- *What are problems you face while needing help regarding diabetes and its medications?*
- *In your personal experience, what are the factors affecting you visiting a clinic in Australia?*
- *What are the enablers while seeking healthcare in Australia?*
- *Was there anything that you liked or didn’t like?*

**Question 6:** What are your thoughts about the level and amount of information that healthcare professionals provide/give to you about your diabetes?

**Prompts;**

- *Do you feel that it is easy or difficult to understand?*
- *Do you feel that you need more or less information?*

**Question 7:** What are your thoughts about your knowledge about diabetes and its medications?

**Prompts;**

- *Inadequate knowledge*
- *Do you think you need more information*
- *Is there anything in particular that you are interested in knowing about (diabetes or medications or both)*
- *Do you think that you manage your diabetes well?*

**Question 8:** What have your experiences with diabetes management been while you have been living in Australia?

**Prompts;**

- *In your experience what are the major differences in India and in Australia*
- *How do you seek help?*

**Question 9:** What does health or wellbeing mean to you? What do you do to keep yourself as healthy as possible, considering that you have diabetes?

**Prompts;**

- *Please tell me what you do in terms of food intake, types of food you eat; in terms of exercise to keep blood sugar normal*
- *Easy or hard to manage as compared to India, why?*

**Question 10:** How do you manage to take medications as per prescribed by your physician?

**Prompts;**

- *Based on your experience, please suggest strategies that you think might help to improve medications adherence?*

**Question 11:** Do you or have you used any Indian traditional medicines to control your blood sugar? Why? How have they worked?

**Prompts;**

- *In Ayurveda, Yoga and Naturopathy, Unani, Siddha and Homoeopathy (AYUSH), which system you follow and why?*
- *In your opinion what are the reasons that you and your family consider to use this medicine. (more effective, safe, less expensive and side effects, better to control blood sugar or permanent cure)*
- *How often do you take them?*
- *How you know about these medicines and from where do you get them?*
- *Have you / do you inform your doctor about these medicines? Reason?*

**Question 12:** What are your thoughts about spiritual health? How do you believe it impacts or influences your diabetes or what you do about your diabetes?

**Prompts;**

- *What is your personal experience or views about meeting a spiritual healer like a priest (Hindu), imam (islam), father (Christian), darbar sahib (sikhs) etc or traditional healers or religious leaders to seek prayers*
- *What is the reason for getting this help?*

**Question 13:** What are your religious beliefs about insulin and other medications? How do these beliefs impact your diabetes management?

**Prompts;**

- *Bovine insulin (cow)*
- *Porcine insulin (pork)*
- *Haram and halal*
- *Neutral (no effect)*

**Question 14:** How do you manage your blood sugar during religious fasts?

**Prompts;**

- *Based on your personal experience, please suggest strategies that you used to manage diabetes during fasting? (It’s difficult or easy, diet control, managing side effects, Fasting is good to control diabetes?, feel weak?)*
